# LRRK2 G2019S Mutated iPSC-Derived Endothelial Cells Exhibit Increased α-Synuclein, Mitochondrial Impairment, and Altered Inflammatory Responses

**DOI:** 10.3390/ijms252312874

**Published:** 2024-11-29

**Authors:** Tuuli-Maria Sonninen, Sanni Peltonen, Jonna Niskanen, Riikka H. Hämäläinen, Jari Koistinaho, Šárka Lehtonen

**Affiliations:** 1 A.I. Virtanen Institute, University of Eastern Finland, 70211 Kuopio, Finland; tuuli-maria.sonninen@uef.fi (T.-M.S.); sanni.peltonen@uef.fi (S.P.); jonna.niskanen@uef.fi (J.N.); riikka.martikainen@uef.fi (R.H.H.); 2 Helsinki Institute of Life Science, University of Helsinki, 00014 Helsinki, Finland; jari.koistinaho@helsinki.fi; 3 Drug Research Program, Division of Pharmacology and Pharmacotherapy, University of Helsinki, 00014 Helsinki, Finland; 4 Neuroscience Center, University of Helsinki, 00014 Helsinki, Finland

**Keywords:** endothelial cell, blood–brain barrier, Parkinson’s disease, iPSC cells, inflammation, MEG3

## Abstract

The blood–brain barrier (BBB) serves as an interface between the bloodstream and the central nervous system. It limits the movement of molecules and immune cells, regulates the entry of nutrients, and removes waste products from the brain. The dysfunction of the BBB has been identified in Parkinson’s disease (PD) but the role of the BBB and endothelial cells (ECs) has not been well studied. *LRRK2* G2019S mutation is the most common PD causing mutation with similar pathophysiology than in sporadic cases. How the mutation affects EC function has not been investigated previously in patient cells. In the study, we used iPSC-derived ECs from PD patients with the *LRRK2* mutation as well as cells from healthy individuals. We report that PD patients’ ECs have higher levels of α-synuclein and an decreased maximal and ATP-linked respiration and altered response to inflammatory exposure, especially to TNFα. In addition, transcriptomic analysis showed upregulation of fatty-acid-synthesis-related pathways in PD patients’ ECs and the downregulation of lncRNA *MEG3*, both of which have been associated with PD. Altogether, PD patients’ ECs manifest some of the PD-related hallmarks and are likely to contribute to the pathogenesis of PD.

## 1. Introduction

Parkinson’s disease (PD) is the fastest-growing human neurological disease due to the aging population, and it is predicted to affect almost 2.5 million people in Europe by 2040 [1]. It is characterized by the loss of dopaminergic neurons in the substantia nigra pars compacta and the presence of Lewy bodies [2]. The loss of dopaminergic neurons is considered to be the reason for motor symptoms (tremor, rigidity, and slowness with walking), but there are several non-motor symptoms that frequently occur and may precede the onset of the motor symptoms. Although most of the PD cases are sporadic, around 10% have a familial background. So far 20 disease-associated genes have been identified, including mutations in *LRRK2*, *SNCA*, *PINK1*, *PRKN*, and *GBA* [3]. The G2019S mutation in *LRRK2* (leucine-rich repeat kinase 2) accounts for 5–13% of the familial cases [4]. This mutation results in excessive activation of the kinase domain, and the clinical pathophysiology closely mimics that of sporadic cases. LRRK2 has been shown to be expressed and is known to be localized in mitochondria, endoplasmic reticulum, Golgi apparatus, the endolysosomal system, and synaptic vesicles. The physiological function of LRRK2 has been linked to neurite outgrowth, vesicle trafficking, the regulation of the autophagy pathway, cytoskeleton dynamics, mitochondrial function, mRNA translation, and the immune system.

While several molecular mechanisms are identified in PD, including α-synuclein pathology, neuroinflammation, mitochondrial dysfunction, and impaired protein degradation, the exact cause remains unknown [5]. Furthermore, disruption of the blood–brain barrier (BBB) has been identified in PD patients and in animal models [6]. The BBB is a semipermeable membrane that separates the central nervous system from the periphery. The BBB is mainly formed by brain endothelial cells (ECs), pericytes, and astrocytes that, together with neurons and microglia, present a functional unit called the neurovascular unit. The BBB maintains the brain homeostasis, regulates the delivery of oxygen and important nutrients to the brain, protects the brain from changes in the periphery, and removes carbon dioxide and toxic metabolites from the brain. Altogether, proper function of the BBB is crucial for the maintenance of healthy brain tissue.

Previous studies have identified decreased P-glycoprotein expression, the increased leakiness of the BBB in basal ganglia, the accumulation of serum proteins, and EC degeneration in PD patients [7,8,9,10,11,12,13]. Animal models of 6-hydroxydopamine (6-OHDA) and 1-methyl-4-phenyl-1,2,3,6-tetrahydropyridine (MPTP) have identified BBB leakage, increased P-gp immunoreactivity, and the infiltration of immune cells [14,15,16,17]. *LRRK2* G2019S mutation has also been shown to increase VCAM1 expression and monocyte attachment in HUVECs [18]. Moreover, the activation of astrocytes in PD can enhance the release of pro-inflammatory cytokines, increasing neuronal death and affecting the function of the BBB [19]. Although BBB impairment is recognized in PD, there have been limited investigations on this topic, and the underlying mechanism is incompletely understood.

Here, we report for the first time how the *LRRK2* G2019S mutation affects human induced pluripotent stem cell (hiPSC)-derived ECs. The impact was examined at the transcriptome and functional levels under basal and inflammatory-exposed conditions.

## 2. Results

### 2.1. Differentiation and Characterization of hiPSC-Derived Endothelial Cells

**Figure 1:**
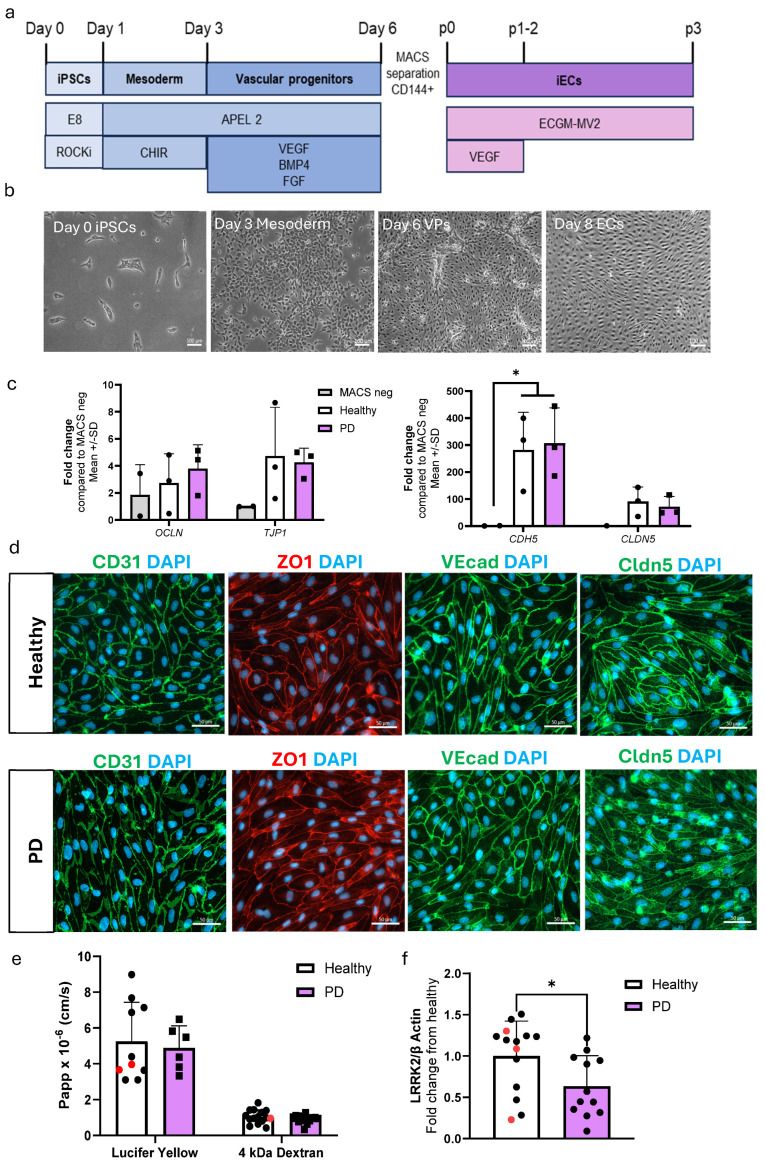
Differentiation and characterization of hiPSC-derived endothelial cells. (**a**) A schematic illustration of the differentiation of hiPSC-ECs. HiPSCs are differentiated first to mesoderm and then vascular progenitors. VE cadherin (CD144) positive cells are MACS separated and ECs are expanded before experiments. (**b**) Representative bright field images of different stages of EC differentiation. Scale bar 100 µm. (**c**) Relative gene expression of *CDH5*, *CLDN5*, *OCLN*, and *TJP1* in ECs compared to CD144 negative cells from MACS separation. *n* = 2 (MACS neg), 3 (healthy), 3 (PD LRRK2), Two-way ANOVA. (**d**) Representative fluorescent images of ECs stained with CD31, ZO1, VE cadherin, and claudin 5. Nuclei stained with DAPI. Scale bar 50 µm. (**e**) Permeability (Papp) of 4 kDa Dextran and Lucifer yellow. *n* = 8–14 (healthy), 1–2 (isogenic, marked as red), 6–13 (PD LRRK2) from four (4 kDa Dextran) or two (Lucifer yellow) independent experiments, Unpaired *t*-test (**f**) Western blot analysis of LRRK2 normalized to β-actin protein levels. *n* = 12 (healthy), 3 (isogenic), 12 (PD LRRK2), Unpaired *t*-test All data shown as mean ± SD. * *p* < 0.05.

The differentiation of ECs was adopted from a previously published protocol [20] with minor modifications. ECs were differentiated to vascular progenitor cells via mesoderm. The progenitors were then sorted and cultured for two passages before being used for experiments (Figure 1a,b). The expression levels of EC-related genes *CDH5*, *CLDN5*, *OCLN*, and *TJP1* were compared to the negative fraction obtained from the MACS separations (Figure 1c). Both healthy and PD ECs exhibited elevated expression levels in the genes of interest in comparison to the MACS negative fraction. Healthy and PD ECs also expressed CD31, ZO1, VE cadherin, and claudin 5 at the protein level (Figure 1d, Figure S1). To evaluate the barrier formation of the ECs, we carried out permeability tests with 4 kDa dextran and Lucifer yellow (LY, 0.4 kDa). ECs showed size selectiveness as permeability was lower for 4 kDa dextran than 0.4 kDa LY (Figure 1e). There were no observed differences between healthy and PD ECs. LRRK2 protein expression was confirmed in ECs using Western blot, and interestingly the levels were decreased in PD ECs (Figure 1f and Figure S2).

### 2.2. LRRK2 Mutation Induces Moderate Changes in Transcriptomics of Endothelial Cells

**Figure 2:**
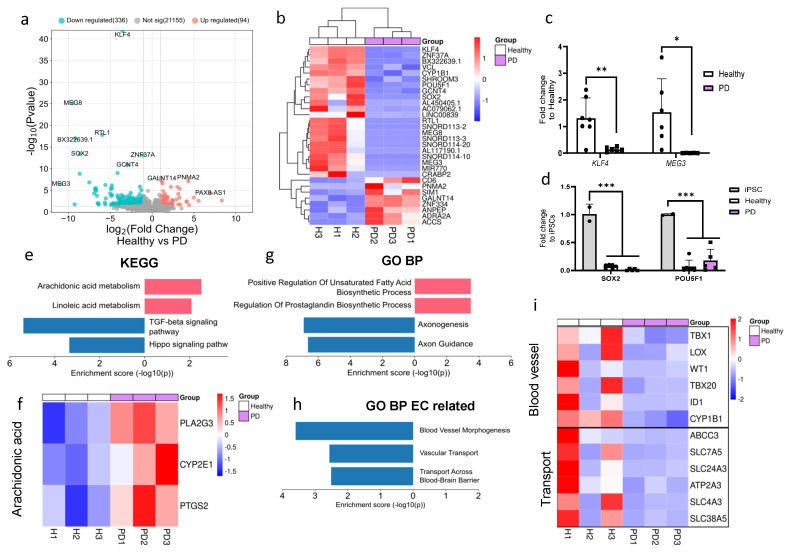
RNA expression analysis of hiPSC-derived endothelial cells from healthy donors and PD patients. (**a**) Volcano plot of up- and downregulated DEGs between healthy and PD ECs based on *p*-value (<0.05) and absolute log2 Fold > 1. (**b**) Cluster heatmap of the top 30 DEGs between healthy and PD ECs based on the *p*-value (<0.05). (**c**) Gene expression levels of *POU5F1* and *SOX2* in healthy and PD ECs compared to hiPSCs, assessed by qPCR. *n* = 2 (hiPSCs), *n* = 6 (healthy), *n* = 5 (PD LRRK2), two-way ANOVA. (**d**) Gene expression of *KLF4* and *MEG3* in healthy and PD ECs quantified with qPCR compared to healthy. *KLF4 n* = 7 (healthy), *n* = 6 (PD LRRK2), *MEG3 n* = 6 (healthy), *n* = 5 (PD LRRK2). Unpaired *t*-test. (**e**) KEGG pathway of up- and downregulated genes in PD ECs compared to healthy. (**f**) Heatmap showing genes related to arachidonic acid metabolism in healthy and PD ECs. (**g**) GO pathway of up- and downregulated genes in PD ECs compared to healthy. (**h**) GO biological processes related to vascular or EC function of up- and down-regulated genes in PD ECs compared to healthy. (**i**) Heatmap showing genes related to blood vessel morphogenesis and vascular transport/transport across BBB in healthy and PD ECs. KEGG, the Kyoto Encyclopedia of Genes and Genomes; GO BP, gene ontology biological processes; PD1–3, patients carrying a mutation in LRRK2 gene (G2019S.) Data shown as mean ± SD. * *p* < 0.05, ** *p* < 0.01, *** *p* < 0.001.

RNA sequencing was conducted to investigate the impact of the *LRRK2* mutation on the transcriptome of ECs. Due to the limited sample size, *p*-value < 0.05 was used to identify differentially expressed genes (DEGs). A total of 336 genes were downregulated and 96 genes were upregulated in PD ECs when compared to healthy controls (Figure 2a). Among the top downregulated DEGs in PD ECs were pluripotency genes (*KLF4*, *SOX2*, and *POU5F1*), long noncoding RNAs (*MEG3*, *MEG8*), and EC-function-associated genes (*KLF4*, *VCL*, *CYP1B1*) (Figure 2b). To validate the expression levels of downregulated genes in ECs, we employed qPCR and confirmed that the levels of *KLF4* and *MEG3* were significantly decreased when compared to healthy ECs (Figure 2c). We also confirmed that the expression of pluripotency genes *SOX2* and *POU5F1* was significantly lower in ECs compared to hiPSCs (Figure 2d). Up- and down-regulated DEGs were analyzed with Enrichr to identify the pathways that were altered between healthy and PD ECs. The pathway analysis (KEGG) revealed that the upregulated genes in PD ECs were involved with the metabolic processes of arachidonic and linoleic acid (Figure 2e,f). The downregulated genes were found to be linked to the TGFβ and hippo signaling pathways (Figure 2e). The upregulated gene ontology (GO) biological processes in PD ECs included the positive regulation of unsaturated fatty acid and prostaglandin biosynthesis (Figure 2g). This finding was consistent with results of the KEGG analysis. The processes that were downregulated were specifically associated with axonogenesis and axon guidance (Figure 2g). We also examined if EC-function-related pathways were altered. In PD ECs, blood vessel morphogenesis and vascular transport/transport across the BBB were reduced (Figure 2g,i).

### 2.3. LRRK2 Mutant Endothelial Cells Have Higher Levels of α-Synuclein

**Figure 3:**
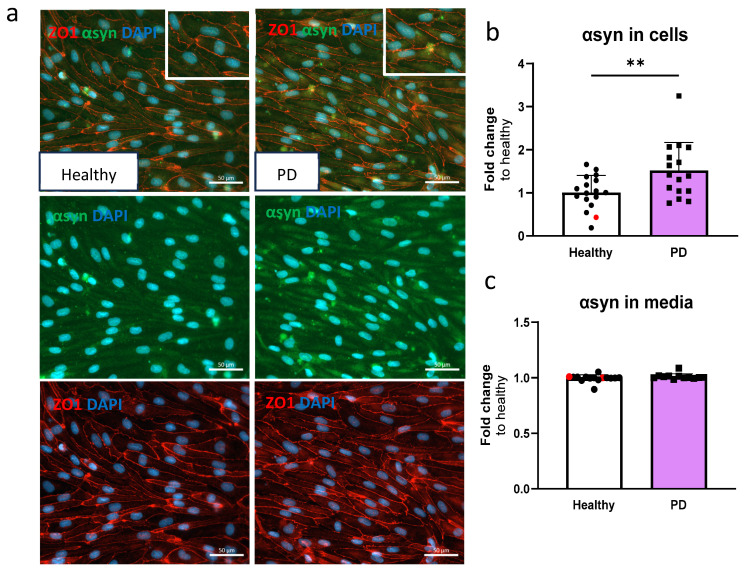
Alpha-synuclein protein expression is increased in LRRK2 mutant endothelial cells. (**a**) Representative fluorescent images of ECs stained with ZO1 and α-synuclein (αsyn). Nuclei stained with DAPI. Scale bar 50 µm. (**b**) Protein expression of α-synuclein in ECs measured with ELISA. Data shown as fold change to healthy + isogenic (marked as red) and results are normalized to total protein amount. *n* = 16 (healthy), 1 (isogenic, marked as red), 14 (PD LRRK2), from five independent experiments. (**c**) Released amount of α-synuclein in media measured with ELISA. Data shown as fold change to healthy + isogenic. *n* = 14 (healthy), 2 (isogenic, marked as red), 12 (PD LRRK2) from five independent experiments. Data shown as mean ± SD. Unpaired *t*-test, ** *p* < 0.01.

Given that α-synuclein accumulation is one of the hallmarks of PD, we quantified the amounts of α-synuclein in ECs. The presence of α-synuclein was detected in ECs by immunostaining (Figure 3a) and measured using ELISA. The amount of α-synuclein in LRRK2 mutant ECs was significantly higher compared to the healthy cells (Figure 3b). No differences in secreted α-synuclein were observed in the media (Figure 3c).

### 2.4. LRRK2 Mutant Endothelial Cells Have Decreased Maximal and ATP Linked Respiration

**Figure 4:**
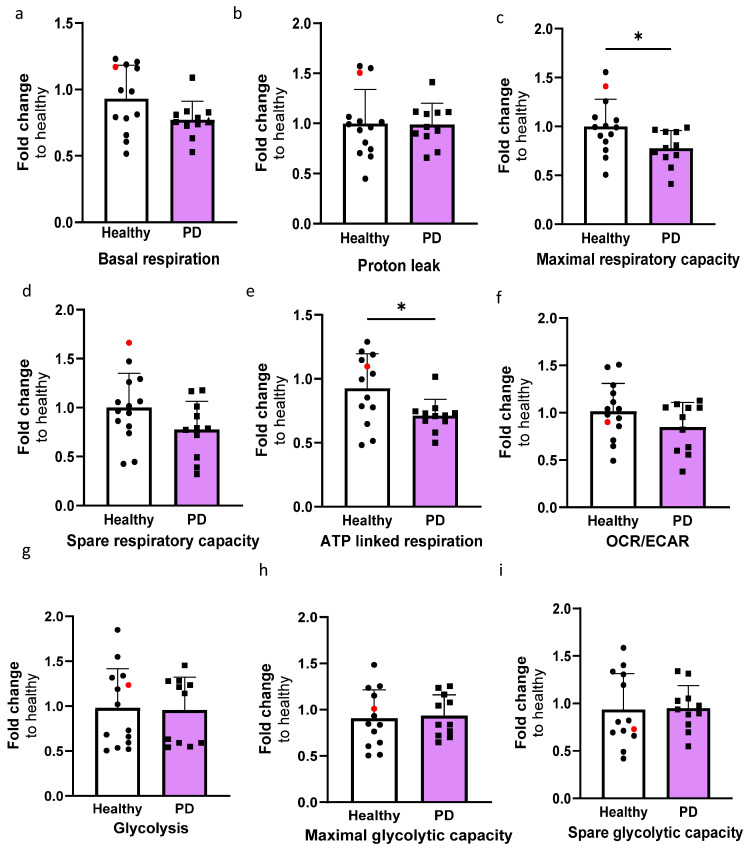
The altered metabolic profile in PD endothelial cells. Oxygen consumption rate (OCR) and extracellular acidification rate (ECAR) were measured with Seahorse XF assay. (**a**–**e**) Calculated OCR values of basal respiration, proton leak, maximal respiration, spare respiratory capacity, and ATP linked respiration, respectively. (**f**) OCR/ECAR ratio from basal level. (**g**–**i**) Calculated ECAR values of glycolysis, maximal glycolytic capacity, and spare glycolytic capacity, respectively. Data shown as fold change to healthy + isogenic. *n* = 11–12 (healthy), 1 (isogenic, marked as red), 10–11 (PD LRRK2) from five independent experiments. Data shown as mean ± SD. Unpaired *t*-test, * *p* < 0.05.

In order to investigate the mitochondrial function of ECs, the oxygen consumption rate (OCR) and extracellular acidification rate (ECAR) were quantified using the Seahorse XF assay (Figure S3). The OCR values for basal respiration, maximal, and spare respiratory capacity, proton leak, and ATP-linked respiration were determined (Figure 4a–e). The levels of maximal respiration and ATP-linked respiration were significantly decreased in PD ECs (Figure 4c,e). A declining pattern was also noted in basal respiration and spare respiratory capacity; although, the differences were not significant (Figure 4d,e). The proton leak remains constant in both healthy and PD ECs. The OCR/ECAR ratio computed from the basal level exhibited a minor decrease in PD ECs but not significantly (Figure 4f). There were no observed differences in glycolysis, maximal glycolytic, or spare glycolytic capacity between healthy and PD LRRK2 groups (Figure 4g–i).

### 2.5. RNA Transcriptomics Reveal More Prominent Effect in PD Endothelial Cells After TNFα Exposure

We used RNA sequencing to compare the impact of 4 h of TNFα and TNFα+IL-1β exposures on the transcriptomics of healthy and PD ECs. Both healthy and PD ECs responded to inflammatory stimuli; however, the reaction was more pronounced when they were exposed to a combination of stimuli (Figure S4a–i). For both exposures, pathway analysis revealed a distinct increase in inflammatory-related pathways and processes, including TNF and NF-κB signaling pathways as well as inflammatory response (Figure S4c,e,h,i). While both healthy and PD ECs showed a comparable response to exposures, the number of DEGs was greater in PD ECs (Figure S4a,f). This phenomenon was particularly observed in cells exposed to TNFα, suggesting a modified response in cells affected by PD.

**Figure 5:**
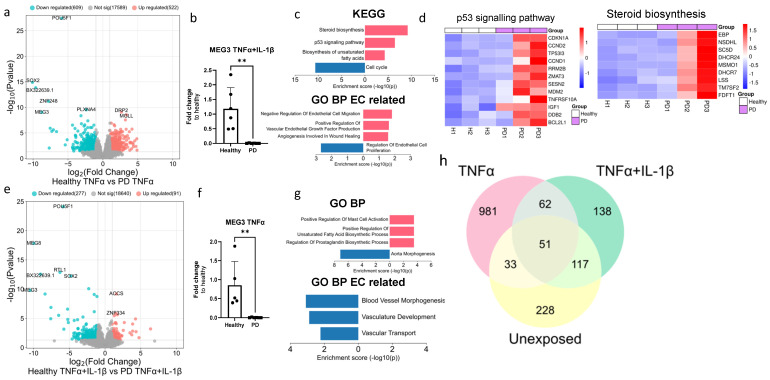
Transcriptional profile of PD endothelial cells after inflammatory stimuli. (**a**) Volcano plot of up- and downregulated DEGs between healthy and PD ECs after TNFα exposure based on *p*-value (<0.05) and absolute log2 Fold > 1. (**b**) Gene expression of MEG3 in healthy and PD ECs quantified with qPCR. *n* = 5 (healthy), 6 (PD LRRK2). Two independent experiments. (**c**) KEGG and GO pathways of up- and downregulated DEGs in PD ECs compared to healthy. (**d**) Heatmap showing genes related to the p53 signaling pathway and steroid biosynthesis in healthy and PD ECs. (**e**) Volcano plot of up- and downregulated DEGs between healthy and PD ECs after TNFα+IL-1β exposure based on *p*-value (<0.05) and absolute log2 Fold > 1. (**f**) Gene expression of MEG3 in healthy and PD ECs. *n* = 6 (healthy), 6 (PD LRRK2). Two independent experiments. (**g**) KEGG and GO pathways of up- and downregulated genes in PD ECs compared to healthy. (**h**) Venn diagram of DEGs between healthy and PD ECs in unexposed, TNFα-, and TNFα+IL-1β exposed conditions. All exposures lasted for 4 h, and the concentrations were: 10 ng/mL TNFα or 10 ng/mL TNFα+ IL-1β. Data shown as mean ± SD, *n* = 3. Unpaired *t*-test, ** *p* < 0.01.

Given that the ECs of both genotypes responded to inflammatory stimuli (Figure S4), next we wanted to examine the differences between healthy and PD ECs after being exposed to inflammation. The exposure to TNFα altered the gene expression between healthy and PD ECs. Specifically, 609 genes were found to be downregulated and 522 upregulated (Figure 5a). The pluripotency genes *SOX2* and *POU5F1* were shown to be reduced in PD ECs, but their expression levels were similar to those in nonexposed cells (raw data). As in nonexposed cells, *MEG3* expression was decreased in PD ECs, and this finding was further validated using qPCR (Figure 5b). The KEGG pathway analysis revealed that upregulated genes were connected to steroid biosynthesis, the p53 pathway, and the biosynthesis of unsaturated fatty acids whereas downregulated genes were linked to cell cycle (Figure 5c,d). The GO pathways were associated with cell migration and vascular endothelial growth factor production for upregulated genes and EC proliferation for downregulated genes (Figure 5c). The exposure to TNFα+IL-1β resulted in the upregulation of 91 genes and the downregulation of 277 genes, which was in the same range as in the nonexposed condition (Figure 5e). Once again, pluripotency genes *SOX2* and *POU5F1* were downregulated in PD ECs but the levels were unchanged from the nonexposed cells (raw data). The expression of *MEG3* was found to be downregulated in PD ECs, as it was seen in TNFα and nonexposed cells. The gene expression was confirmed with qPCR (Figure 5f). Altered pathways of upregulated genes were related to mast cell activation, the regulation of unsaturated fatty acid and prostaglandin biosynthesis processes, while GO pathways related to ECs were linked to blood vessel morphogenesis, vasculature development and vascular transport in downregulated genes (Figure 5g). Altogether, the changes between healthy and PD ECs were more prominent with TNFα alone, whereas, the TNFα+IL-1β combination did not lead to additional patient specific changes when compared to the unexposed condition (Figure 5h).

### 2.6. Prolonged Inflammatory Environment Affects Endothelial Cells Viability

**Figure 6:**
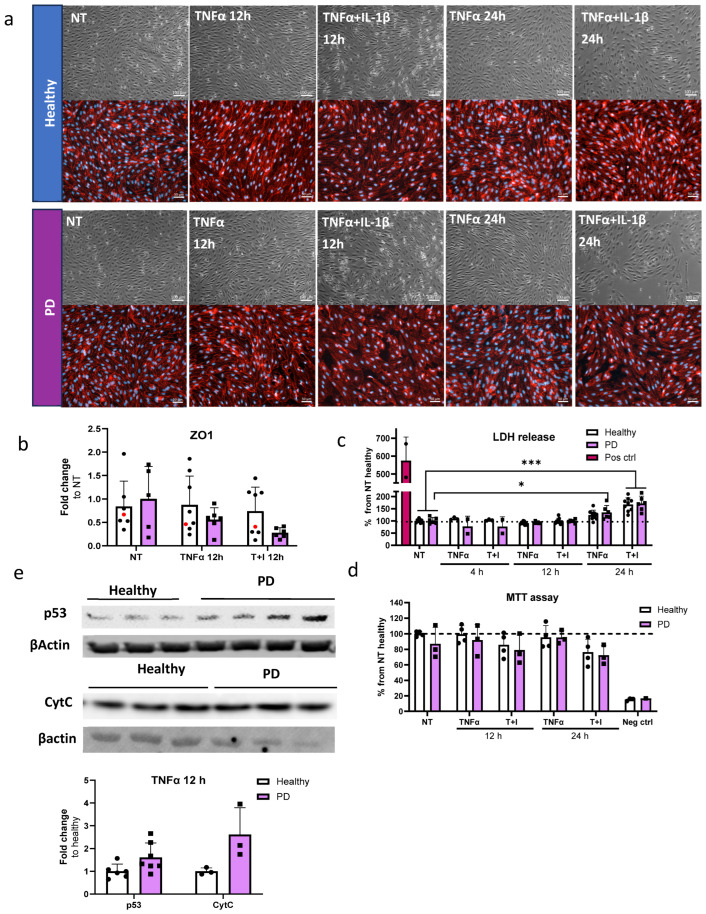
Cell viability and cytotoxicity in endothelial cells after inflammatory stimuli. (**a**) Representative bright field (upper panel) and immunofluorescence images stained for ZO1 (lower panel) of ECs, nonexposed (NT), 12 h TNFα and TNFα+IL-1β exposed, 24 h TNFα and TNFα+IL-1β exposed. Scale bar 50 µm. (**b**) Quantification of the immunocytochemistry. Fluorescence normalized to DAPI. *n* = 6–7 (Healthy), 1 (isogenic),5–6 (PD LRRK2) from two independent experiments. (**c**) LDH release from ECs of non-exposed and after 4, 12 and 24 h TNFα and TNF+IL-1β (T+I) exposure. *n* = 3–9 (healthy), 2–6 (PD LRRK2) from two independent experiments. (**d**) MTT assay of non-treated and 12 and 24 h TNFα and TNF+IL-1β treated ECs. *n* = 2–4 (healthy), 1–3 (PD LRRK2). (**e**) Western blot analysis of p53 and Cytochrome C (CytC) in healthy and PD EC normalized to β-actin protein levels. Representative images and quantification of the data. *n* = 3–6 (healthy), 3–7 (PD LRRK2). Data shown as mean ± SD. Two-way ANOVA with Tukey’s multiple comparison test, * *p* < 0.05, *** *p* < 0.001.

Next, we investigated the cell viability and cytotoxicity in the ECs after exposure to inflammatory stimuli, especially TNFα, which generated alterations in transcription levels related to the p53 signaling pathway. For this, we utilized the longer stimulation times of 12 and 24 h. First, we examined whether longer exposure to inflammatory stimuli affects cell viability. The viability was evaluated with brightfield imaging and subsequent immunostaining for tight junction protein ZO1 (Figure 6a). In PD ECs, TNFα+IL-1β exposure for 24 h resulted in a cell number decrease, while ZO1 expression was disrupted in PD ECs with all exposures. On the other hand, in healthy ECs, ZO1 disruption was only evident in combined exposures. Quantitative analysis of the immunocytochemistry showed a 1.45-fold decrease in ZO1 expression in PD ECs after 12 h of TNFα exposure (Figure 6b). A 1.7-fold decrease was observed after combination exposure of TNFα+IL-1β (Figure 6b). We then assessed the release of LDH, a cytotoxicity marker. Elevated LDH levels by 1.7-fold were observed with 24 h of TNFα+IL-1β exposure in both healthy and PD ECs, as compared to nonexposed cells (Figure 6c). In PD ECs, 24 h of TNFα exposure also significantly increased the LDH release by 1.35-fold which was not seen in healthy ECs. The viability and metabolic activity of cells were assessed using MTT assay (Figure 6d). The combined exposure led to a modest decrease in activity in both healthy and PD ECs compared to the unexposed group; although, the change was not statistically significant. TNFα had no effect on viability.

Transcriptome analysis revealed that PD ECs exhibited the activation of the p53 pathway and a reduction in the cell cycle after being exposed to TNFα for 4 h. In order to determine whether the RNA expression translates to protein levels, p53 and Cytochrome C, a downstream target of p53, expression was analyzed by Western blot for TNFα-exposed cells (Figure 6e and Figure S5). As expected, the expression of p53 and Cytochrome C was elevated in PD ECs.

### 2.7. Endothelial Cells Show Aberrant Phenotype in Pro-Inflammatory Conditions

**Figure 7:**
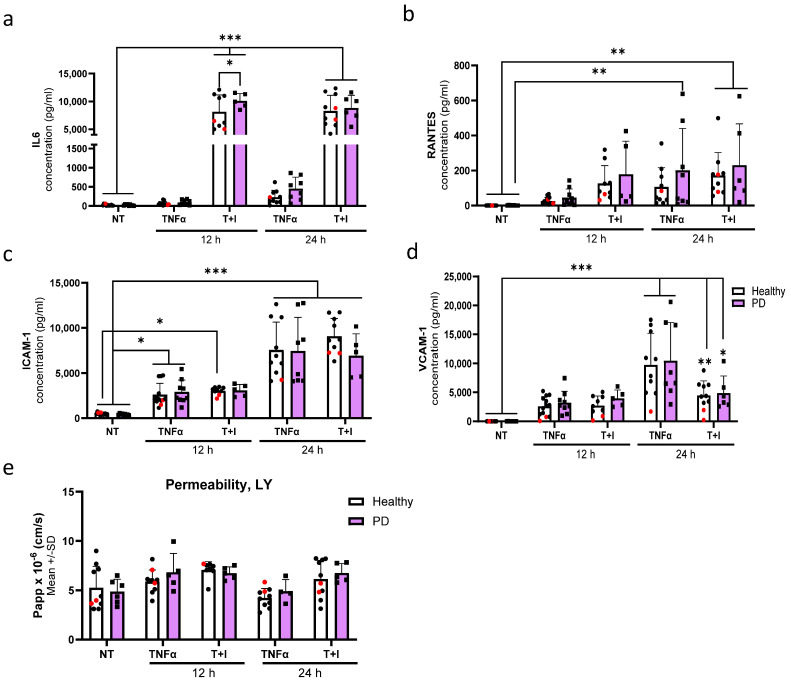
Aberrant phenotype of endothelial cells in pro-inflammatory conditions. (**a**–**d**) Release of (**a**) IL6, (**b**) RANTES, (**c**) ICAM1, and (**d**) VCAM1 quantified from media after 12 and 24 h TNFα and TNFα+IL-1β (T+I) exposures measured with CBA. *n* = 10–13 (healthy), 1–2 (isogenic, marked as red), 5–9 (PD LRRK2) from three independent experiments. (**e**) Permeability for Lucifer yellow (LY) in nonexposed, 12, and 24 h TNFα and TNFα+IL-1β (TI) exposed ECs. *n* = 6–8 (healthy), 1–2 (isogenic), 4–6 (PD LRRK2) from two independent experiments. All data shown as mean ± SD. Two-way ANOVA with Tukey’s multiple comparison test. * *p* < 0.05, ** *p* < 0.01, *** *p* < 0.001.

To assess the effect of inflammatory exposures on the secretion of cytokines from endothelial cells, we first exposed the cells to TNFα or a combination of TI for either 12 or 24 h. IL-6 release was especially increased after combination exposures. At 12 h, the release was significantly higher in PD ECs, with no difference at a later time point when compared to healthy ECs (Figure 7a). An elevation in the release of RANTES was observed at 12 h in both healthy and PD ECs following TI exposure (Figure 7b). This elevation persisted at 24 h with no differences between genotypes, likely due to large variability among the lines that responded. The levels of ICAM1 gradually increased over time. However, various exposures did not have a significant impact on the response of both healthy and PD ECs (Figure 7c). Although, a decrease in ICAM1 levels was observed in PD ECs after 24 h combination exposure. The release of VCAM1 was highest after 24 h of TNFα exposure, but no differences were seen between healthy and PD ECs (Figure 7d). We further investigated the permeability using LY under the same inflammatory stimulating condition (Figure 7e). No changes in permeability were detected between healthy and PD ECs. However, the inflammatory conditions slightly increase the permeability at 12 h; still, this change was not statistically significant.

## 3. Discussion

In the past, PD research concentrated mainly on neurons, but a current interest in studying other types of brain cells has shifted this viewpoint. While the involvement of astrocytes [21,22] and microglia [23] in PD pathogenesis is now well-established, there has been limited research focused on investigating the role of ECs, or the BBB, in this context. A recent study using single cell RNA sequencing from PD and healthy individuals suggested that ECs may have a significant impact on cell–cell communication in PD. The study identified ECs as the core cell cluster involved in intracellular communications [24]. Nevertheless, the effect of PD-related mutations on EC function remains unexplored.

Although the overall impact of the *LRRK2* mutation on the ECs transcriptome was moderate, several noteworthy genes were identified. Kruppel-like factor 4 (KLF4) and maternally expressed gene 3 (*MEG3*) were downregulated in PD ECs. KLF4 has an important role in regulating several biological processes, including embryogenesis, proliferation, differentiation, neuroinflammation, oxidative stress, and apoptosis [25]. In ECs, KLF4 has been shown to promote vascular integrity and maintain vascular health [26]. In stroke models, KLF4 has a protective effect on microvascular ECs [27,28]. In addition, KLF4 has been shown to regulate EC activation after pro-inflammatory stimuli. Moreover, the overexpression of KLF4 induces the expression of several anti-inflammatory and anti-thrombotic factors [29]. In contrast to ECs, the knockdown of KLF4 has shown beneficial effects on neuronal survival in several PD models [25]. This indicates that KLF4 has different roles in various cell types and downregulation might have a negative effect on LRRK2 mutated ECs.

*MEG3* is a long noncoding RNA, and its role has been studied especially in cancer. Recent contradicting studies have identified altered MEG3 levels in PD patients, two studies reported decreased levels in patient plasma, and one reported an increase levels [30,31,32]. Huang et al. studied the effect of *MEG3* in SH-SY5Y cells treated with MPP+ [30]. The overexpression of *MEG3* increased LRRK2 expression, improved cell viability, and decreased apoptosis, indicating a connection between *MEG3* and LRRK2. In addition, decreased expression of *MEG3* was found in pericytes with *LRRK2* G2019S mutation [33]. In ECs, *MEG3* has been shown to prevent vascular EC senescence, control VEGFA-mediated angiogenesis, and regulate proliferation and migration [34,35,36]. The knockdown of *MEG3* in HUVECs decreased cell viability, increased apoptotic cell rate, and impaired migration function [37]. Additionally, *MEG3* knockdown induced cellular senescence, characterized by increased senescence-associated β–galactosidase activity, increased levels of endogenous superoxide, impaired mitochondrial structure and function, and impaired autophagy [38]. Taken together, these studies indicate that *MEG3* has an important role in EC function and that the loss of expression in PD cells could be linked to pathogenesis.

The accumulation of α-synuclein is one of the hallmarks of PD. α-synuclein is a small 14 kDa protein highly expressed in neurons but also expressed in peripheral cells and ECs [6]. It can be transported bi-directionally across the BBB. Higher plasma levels of exosomal α-synuclein have been found in PD patients, and post-mortem studies have associated α-synuclein aggregates with endothelial degeneration and decreased P-gp expression [9,39,40]. Increased α-synuclein levels have also been detected in neurons and astrocytes with *LRRK2* G2019S mutation [21,22,41]. Our findings demonstrate that LRRK2 mutant ECs have higher levels of α-synuclein, but the released levels remained the same compared to healthy cells. This could indicate failure in exporting or the degradation of α-synuclein in PD ECs; although, this would require more extensive studies.

The transcriptomic analysis revealed alterations in pathways related to unsaturated fatty acids and their biosynthesis in PD ECs. Fatty acids have several important roles in the body, as they serve as a source of energy, are major components of cell membranes, and regulate inflammatory responses [42]. The excessive intake of fatty acids is associated with obesity, energy overload, and increased brain inflammation. Polyunsaturated fatty acids can exert a direct cytotoxic effect on α-synuclein. Polyunsaturated fatty acids can bind to α-synuclein and promote oligomerization, and high concentrations of DHA increase the intracellular accumulation of soluble and insoluble α-synuclein and neuronal injury in A53T mice [43,44,45]. In addition, a higher intake of arachidonic acid has also been linked to an increased risk of PD pathogenesis [46]. Besides fatty acids themselves, fatty acid binding proteins (FABPs) 3 and 5 have been linked to α-synuclein accumulation and aggregation, mitochondrial dysfunction, and increased apoptosis [42]. The RNA expression of both genes was elevated in PD ECs after TNFα exposure. The increased expression of FABPs combined with increased fatty acid biosynthesis might exacerbate the toxic effect of α-synuclein in ECs.

Mitochondrial dysfunction is one of the key mechanisms linked to PD pathogenesis. Higher oxidative stress, reduced mitochondria membrane potential, decreased ATP production, mitochondria DNA damage, elongated mitochondria, mitochondrial fragmentation, and mitophagy have been observed in postmortem human tissues and animal and cellular models of LRRK2 [47]. PD ECs with the *LRRK2* mutation showed altered mitochondrial function, including decreased maximal respiration and ATP-linked respiration. Alterations in oxygen consumption have been found in other cell types, including hiPSC-derived neurons [48] and astrocytes [21] which carry the *LRRK2* G2019S mutation. This could indicate a similar effect of the mutation, regardless of the cell type.

One common hallmark of several neurodegenerative diseases, including PD, is neuroinflammation. The effect of inflammatory stimuli on PD ECs was studied on a transcriptomic and functional level. While TNFα+IL-1β exposure elicited a more pronounced response in both healthy and PD ECs compared to TNFα alone, the combination did not cause a substantial genotypic difference in transcriptomics. This could suggest that PD ECs have an altered response only to specific stimuli. At the functional level, the *LRRK2* mutation did not cause major differences. ECs responded to inflammatory exposures by increasing the release of cytokines, but the variation across different cell lines was substantial. This could suggest that the response is individual rather than LRRK2-related. Interestingly we did detect increased IL6 secretion after 12 h TNFα+IL-1β exposure. Our previous study with *LRRK2* mutated astrocytes also showed increased secretion of IL6 after inflammatory stimuli [21]. Moreover, elevated levels of IL6 have been detected in PD patients and linked to disease mortality [49,50,51,52].

Upon longer inflammatory exposure, cell death was increased in ECs. This was seen in both healthy and PD ECs after 24 h of combination exposure, but already in PD ECs after 24 h of TNFα exposure. We also detected elevated levels of p53 and Cytochrome C in PD ECs after 12 h of TNFα exposure. The p53 signaling pathway was also linked to upregulated genes in PD ECs after 4 h TNFα exposure. P53 is involved in several processes which are related to PD, like neuronal oxidative stress, apoptosis, and abnormal protein aggregation [53]. Moreover, high expression of p53 has been found in PD cell models and in the substantia nigra region in PD patients and animal models [54,55,56]. In addition, p53 can induce apoptosis through mitochondrial cytochrome C release and the activation of caspases [57]. Interestingly, *MEG3* has been shown to regulate p53 signaling, but the response seems to be cell-specific. For example, in neurons, *MEG3* activates p53 and mediates neuronal death in stroke [58]; whereas, in cardiac fibroblasts, *MEG3* does not alter the p53 response [59]. The role of *MEG3* and p53 in HUVECs was studied by Ali et al. [60]. After 4 h of TNFα exposure, *MEG3* knockdown induced p53 activation by increasing the expression of p53 target genes, promoting apoptosis, and inhibiting proliferation. Whether LRRK2 mutation is linked to *MEG3* and p53 activation is still unclear and would need further studies.

*LRRK2* G2019S mutation is known to increase the kinase activity [61] but how the mutation affects the expression of LRRK2 has not been studied with ECs. Here we observed decreased levels of LRRK2 in PD ECs. Considering that we did observe only some PD related hallmarks in PD ECs, it is possible that ECs lower their mutation load by downregulating the expression of LRRK2. In turn, we detected a more robust effect in PD ECs after inflammatory stimuli especially affecting transcription and cell viability. Inflammatory exposure has been shown to increase the expression of LRRK2 in HUVECs with G2019S mutation [18]. Although not studied here, if this happened in PD ECs it would suggest that during additional stress, the increased mutation load could lead to more detrimental effects.

### Limitations of the Study

This study focused on the *LRRK2* G2019S mutation in ECs only. It is important to keep in mind that these results represent LRRK2-related cases and may not directly translate into sporadic ones. We have not checked additional mutations from the *LRRK2*-mutated cells, and there is a small possibility that additional mutations or risk variants might influence the results. Although the dysfunction of the BBB and ECs is recognized in PD, its role probably is not as substantial as, for example, in Alzheimer’s disease, and individual variation might play a part. Our results showed variation between healthy and PD individuals especially in transcriptomics and inflammatory responses. Of course, a low number of cell lines could mask the differences and ideally, more lines should be included. One way to reduce the variation is to use isogenic lines. We included one isogenic line, but this was absent from the transcriptomic studies. It would be important to repeat the RNA sequencing with additional lines and isogenic controls. Additionally, we only used 2D monoculture, and the remaining BBB cell types were missing. The BBB is a multicellular unit, including astrocytes and pericytes, and their presence could change the properties of ECs and, thus, also the effect of the *LRRK2* mutation. Within this study, we used a protocol for deriving vascular ECs as opposed to brain ECs. Because of this, some BBB-specific changes might have been overlooked in our study. Given our emphasis on inflammation, we aimed to ensure that cells respond to inflammatory stimuli. While there are existing protocols published for obtaining brain-like ECs, these protocols have major limitations. It has been shown that brain-like EC protocols produce cells with more epithelial cell phenotypes and lack the EC-specific response to inflammatory stimuli [62,63,64]. Furthermore, one common feature of all hiPSC ECs is the absence of P-glycoprotein expression and, more importantly, its functionality [65]. Similarly, we observed very low levels in our cells. The decreased expression of P-glycoprotein has been linked to PD and the investigation of this would require a different model system. Advances in different 3D models, like organ-on-chips or vascularized brain organoids, could provide more accurate models in the future.

## 4. Materials and Methods

### 4.1. Cell Culture

Human iPSC (hiPSC) lines used in this study included six healthy controls, three lines with G2019S mutation in the *LRRK2* gene and one isogenic line (Table S1). Healthy lines 4–6 and PD LRRK2 line 1 were generated and characterized in the Stem Cell Laboratory, at the University of Eastern Finland [66]. Healthy lines 1–3 were obtained from Takara Bio, Kusatsu, Japan (Y00275, Y00305, and Y00325) and PD LRRK2 lines 2 and 3 and the isogenic line were from NIBSC.

### 4.2. Endothelial Cell Differentiation

Two differentiation protocols were used in this study (referred to as protocols 1 and 2). Detailed information of lines and protocols used in different experiments are listed in Table S2. In both protocols, ECs were differentiated first into EC progenitors and then vascular ECs with small differences in small molecules. Protocol 1 was more efficient and for that reason it was chosen for most of the experiments. Protocol 2 was used to measure α-synuclein levels with ELISA and metabolic function with Seahorse assay.

For protocol 1, ECs were differentiated according to the protocol published by Harding et al. [20] with minor modifications. To start the differentiation, hiPSCs were plated to Matrigel (Corning, New York, NY, USA)-coated dishes at a density of 15–25 k/cm^2^ in E8 media (Gibco, Waltham, MA, USA) with 5 µM of ROCK inhibitor (Y-27632 dihydrochloride, Selleckchem, Houston, TX, USA). The next day (day 1), the media was changed to Stemdiff APEL2 media (Stemcell Technologies, Vancouver, BC, Canada) supplemented with 6 µM CHIR-99021 (Cayman Chemical Company, Ann Arbor, MI, USA). On day 3, the media was changed to APEL2 media with an additional 25 ng/mL of BMP4 (Peprotech, Waltham, MA, USA), 10 ng/mL of FGF (Peprotech), and 50 ng/mL of VEGF (Peprotech). Cells were cultured in this medium for three days, changing media on day 5. On day 6, ECs were sorted by magnetic separation (Miltenyi Biotec, Bergisch Gladbach, Germany) using CD144 microbeads according to the manufacturer’s protocol. CD144 positive cells were plated on Matrigel-coated dishes in Endothelial Growth Media MV2 (ECGM-MV2, PromoCell, Heidelberg, Germany) with an additional 20 ng/mL of VEGF for the first passage. ECs were expanded, and the cells in passage three were used for the experiments.

For protocol 2, ECs were differentiated using a protocol published by Patch et al. [67]. Briefly, the hiPSCs were dissociated using EDTA (Invitrogen, Waltham, MA, USA) and seeded at a density of 300,000 cells/35 mm plate in E8 medium supplemented with ROCK inhibitor. The next day, the induction of the lateral mesoderm was started by replacing the media with N2B27 medium supplemented with 8 µM CHIR-99021 and 25 ng/mL BMP4 and continued for three days. The EC induction was started by replacing the N2B27 media with StemPro-34 SFM medium (Life Technologies, Waltham, MA, USA) supplemented with 200 ng/mL VEGF and 2 µM Forskolin (Sigma, Burlington, MA, USA). The cells were incubated in StemPro medium for two to four days, changing the medium every day. When the areas of differentiation were visible, the ECs were sorted by magnetic separation using CD144 microbeads according to the manufacturer’s protocol. CD144 positive cells were plated on 6 cm Matrigel-coated plates in StemPro medium supplemented with 20 ng/mL VEGF.

### 4.3. Immunocytochemistry

ECs were fixed with 4% formaldehyde (VWR, Radnor, PA, USA) for 15 min at room temperature (RT) or methanol (VWR, Radnor, PA, USA) for 15 min at 4 °C followed by washes with PBS. For intracellular antigens, the ECs were permeabilized with 0.2% Triton X-100 in PBS (Sigma, Burlington, MA, USA) for 20 min at RT. The cells were blocked with 5% normal goat serum (NGS, Vector Labs, Newark, CA, USA) or 10% horse serum (HS, Gibco, Waltham, MA, USA) in PBS at RT for 1 h and then incubated with primary antibodies in 5% NGS or 10% HS at 4 °C overnight. For unconjugated antibodies, the cells were incubated in 5% NGS or 10% HS in PBS containing a secondary antibody for 1 h at RT. The cells were washed with PBS, and the nuclei were visualized with DAPI (ThermoFisher, Waltham, MA, USA). The cells were imaged with the Zen Observer Z1 or Zen Imager AX10 (Jena, Germany). The antibodies used in this study are listed in Table S3.

### 4.4. RT-qPCR

RNA was extracted using the RNA easy MiniKit (Qiagen, Venlo, The Netherlands) and the RNA concentration was measured with NanoDrop or DS-11 FX Spectrophotometer/Fluorometer (Denovix, Wilmington, DE, USA). An amount of 500–1000 ng of the RNA was converted to cDNA (Maxima Reverse Transcriptase, ThermoFisher, Waltham, MA, USA). RT-qPCR was conducted using the Maxima probe/ROX qPCR master mix (ThermoFisher, Waltham, MA, USA) to quantify the relative expression of genes of interest utilizing TaqMan assays (Table S4) with Step One Plus (Applied Biosciences, Waltham, MA, USA). The Ct mean value was normalized to the internal Ct mean value of β-actin, and the relative expression was presented as a fold change compared to the control group.

### 4.5. α-Synuclein ELISA

The amount of endogenous α-synuclein was determined from ECs using a human α-synuclein ELISA kit (Invitrogen, Waltham, MA, USA and Biolegend, San Diego, CA, USA). For the assay, cells were lysed with M-PER (Invitrogen, Waltham, MA, USA) or cell extraction buffer (Invitrogen, Waltham, MA, USA) and diluted 1 to 10 in reagent diluent. The luminescence or absorbance 450 nm was measured with PerkinElmer’s VICTOR2 multilabel plate reader (Wallac, Turku, Finland). The α-synuclein concentration from the cells was standardized to total protein concentration using the Pierce™ BCA Protein Assay Kit (ThermoFisher, Waltham, MA, USA). Media samples were collected from ECs cultured for three days without media change. Samples were stored at −70 °C before analysis. A human α-synuclein ELISA kit (AssayGenie, Dublin, Ireland) was used to measure the α-synuclein levels from media. The luminescence or absorbance at 450 nm was measured with PerkinElmer’s VICTOR2 multilabel plate reader.

### 4.6. Seahorse

The oxygen consumption rate (OCR) and the extracellular acidification rate (ECAR), indicators of mitochondrial respiration and glycolysis, respectively, were measured in real time from live cells with the Seahorse Extracellular Flux (XF) 24 or 96 Analyzer (Seahorse Bioscience, North Billerica, MA, USA). First, the ECs were plated on Matrigel-coated plates in StemPro34 or ECGM-MV2 medium depending on protocol used and incubated overnight. Before the assay, the medium was changed to Seahorse XF Assay medium (Seahorse DMEM supplemented with 2 mM Glutamax) and incubated for 1 h at 37 °C without CO2 before adding the compounds. The final concentrations of D-glucose and sodium pyruvate were 10 mM and 0.5 mM, respectively. For oligomycin, FCCP, antimycin, and rotenone, the concentrations were 1.0 µM. The assay was performed as follows: 2 min mix time, 2 min wait time, and 3 min measure cycle. After the assay, the results were normalized to protein concentrations using Pierce™ BCA Protein Assay Kit (ThermoFisher). The results were analyzed with the Wave program (Agilent Technology, Santa Clara, CA, USA). Wells with negative values or aberrant OCR or ECAR patterns were excluded from each of the individual experiments.

### 4.7. Inflmammatory Exposures

To assess the inflammatory response, ECs were exposed to TNFα or a combination of TNFα (Peprotech) and IL-1β (Peprotech). Detailed information on duration, concentrations, and assays are listed in Table S5. Briefly, ECs were plated in ECGM-MV2 medium, and the next day, the media was changed to human Endothelial Serum Free Media (ESFM, Gibco, Waltham, MA, USA) with additional 1X B27 (Gibco, Waltham, MA, USA) and 10 ng/mL FGF (Peprotech). The following day, we initiated inflammatory exposures using the same media as described above.

### 4.8. Total RNA Sequencing

Cells were lysed on ice (Buffer RLT + β-mercaptoethanol 10 µL/mL), and RNA was extracted directly after lysis with the Qiagen RNeasy Mini Kit (Qiagen) with DNase I digestion (Qiagen). RNA concentration was measured with DS-11 FX Spectrophotometer/Fluorometer (Denovix). Amount of 1–4 µg of RNA were sent for library preparation and sequencing (performed by Azenta, Burlington, MA, USA). The quality was evaluated with FastQC, and the mean quality score for the samples was 35.30. The library preparation workflow included ribosomal RNA depletion, RNA fragmentation and random priming, cDNA synthesis, end repair, 5′ phosphorylation and dA-tailing, adaptor ligation and PCR enrichment. Sequencing was carried out with Illumina NovaSeq, PE 2 × 150 (Illumina, San Diego, CA, USA). Sequence reads were trimmed by using Trimmomatic v.0.36 to remove possible adaptor sequences and nucleotides of poor quality. Trimmed reads were mapped to the Homo sapiens GRCh38 reference genome available on ENSEMBL using the STAR aligner v.2.5.2b. Unique gene hit counts were calculated by using featureCounts from the Subread package v.1.5.2 and used for downstream differential expression analysis (DESeq2). The Wald test was used to generate *p*-values and log2 fold changes, and the Benjamini–Hochberg for adjusted *p*-values. Genes with a *p*-value < 0.05 and absolute log2 fold change > 1 were called differentially expressed genes (DEGs) for healthy vs. PD LRRK2 and genes with an adjusted *p*-value < 0.05 and absolute log2 fold change > 1 when comparing exposures within the group. Pathway enrichment analysis (KEGG and gene ontology biological processes, GO BP) was conducted on DEGs using Enrichr [68,69,70].

### 4.9. LDH Cytotoxicity Test

To assess the toxicity of TNFα and IL-1β exposures, the release of LDH was measured from EC media samples at different time points (Table S5) using the CyQuant LDH Cytotoxicity assay (Invitrogen, Waltham, MA, USA). The samples were analyzed with PerkinElmer’s VICTOR2 multilabel plate reader.

### 4.10. MTT Assay

ECs were plated to 96-well plate and treated, as previously described (Table S5). After inflammatory exposure, a part of the cells was exposed to 1% Triton-X for 5 min to induce cell death. This was used as a negative control. Then, fresh media was changed with an additional 1.2 mM of 3-(4,5-Dimethylthiazol-2-yl)-2,5-diphenyltetrazolium bromide (MTT) (VWR, Radnor, PA, USA), and cells were incubated for 2 h at 37 °C. After the incubation, media was re-moved, and DMSO was added to the cells and incubated at RT overnight. The next day, 100 µL of sample was transferred to a new 96-well plate, and absorbance was measured at 595 nm with PerkinElmer’s Victor2 multilabel plate reader.

### 4.11. Western Blotting

Protein was extracted from ECs using in-house made RIPA buffer (50 mM Tris, 1% Triton-X 100, 0.5% Sodium deoxycholate, 0.1% SDS, 150 mM NaCl) with Pierce™ protease inhibitor (ThermoFisher). The total protein amount was measured with Pierce™ BCA Protein Assay Kit (ThermoFisher). Samples were mixed with Laemmli sample buffer (60 mM Tris, 10% glycerol, 2% SDS, 1% Bromophenol blue, with 5% 2-Mercaptoethanol added just before use) and incubated at 95 °C for 5 min. An amount of15 µg (P53, Cytochrome C) or 30 µg (LRRK2) of protein was resolved with 12% gels (P53, Cytochrome C) or 4.5–15% gradient gel in Tris-Glycine running buffer.

Proteins were transferred to 0.2 µm PVDF pre-cut membrane transfer pack (Bio-rad, Hercules, CA, USA) and transferred with Trans-Blot Turbo® transfer system (Bio-Rad, Hercules, CA, USA). Membranes were blocked in 5% Milk powder in TBST (20 mM Trish, 150 mM NaCl, 0.1% Tween-20, pH 7.6), and the membranes were incubated overnight at 4 °C with primary antibodies diluted in 5% BSA in TBST (1:1000): p53, (1:1000): Cytochrome C, (1:10,000): LRRK2. Membranes were washed with TBST and incubated with HRP-conjugated anti-bodies for 2 h at room temperature. After washing, detection was performed with Super-Signal™ West Pico PLUS Chemiluminescent Substrate (ThermoFisher, Waltham, MA, USA) and imaged with ChemiDoc™ MP Imaging System (Bio-Rad, Hercules, CA, USA). For normalization, membranes were incubated with anti- β-actin primary antibody (1:1000) diluted in 5% BSA in TBST overnight at 4 °C. Cy3-conjugated secondary antibody was diluted in 1:1000 in TBST and incubated for 2 h at room temperature. After washing, the membrane was imaged with the same equipment, as previously described. The intensity of the target bands was quantified with ImageLab (version 5.1, Bio-Rad Laboratories, Hercules, CA, USA). Detailed information about antibodies is found in Table S3.

### 4.12. Quantification of Immunofluorescence

ECs were fixed and stained as previously described. Images were taken with Zen Imager AX10, a minimum of two images per sample. Fluorescence was analyzed with Fiji (ImageJ, version 2.16.0). The IntDen of each sample was normalized to the number and intensity of DAPI.

### 4.13. Cytometric Bead Assay

As amount of released cytokines was measured with the cytometric bead array (CBA) Human Soluble Protein Master Buffer Kit (BD, Franklin Lakes, NJ, USA). Before analysis, media samples from nonexposed and exposed samples were collected and stored at −70 °C. The capture beads used were: Human IL-6, Human RANTES, Human ICAM1, and Human VCAM1. The samples were analyzed with CytoflexS (Becman Coultier, Brea, CA, USA). At least 300 events per cytokine were measured. Excitation was carried out with 638 nm red laser, and the bead clusters were detected with 660/20 BP (APC) and 780/60 BP filters (APC-A750). Cytokine reporter PE was excited with a 561 nm yellow laser, and a filter 585/42 BP was used for detection. Data were analyzed using FCAP Array 2.0 (SoftFlow, Pécs, Hungary), and cytokine concentrations were calculated by regression analysis from known standard concentrations.

### 4.14. Permeability Assay

ECs were plated to Matrigel-coated 24 well inserts (0.4 µm pore size, Sarstedt, Nümbrecht, Germany) at a density of 100 k/cm^2^ in ECGM-MV2. The next day, the media was changed to human Endothelial Serum Free Media (ESFM, Gibco) with additional 1X B27 (Gibco) and 10 ng/mL FGF (Peprotech). The following day, media was refreshed, or inflammatory exposures were started (Table S5). Permeability was measured with 4 kDa FITC Dextran (Merck, Darmstadt, Germany) and Lucifer yellow (LY, Merck, Darmstadt, Germany)) from unexposed ECs. LY was used to measure permeability from inflammatory stimulated ECs. An amount of 150 µL of dextran (500 µg/mL) or LY (100 µg/mL) solution was added to the apical side and 800 µL of fresh media to the basolateral side. Basal ESFM was used for the assay. Samples were collected from the wells at timepoints of 20, 40 and 60 min and the volume was replaced with fresh media. The fluorescence values were measured with PerkinElmer’s Victor2 multilabel plate reader. The background was reduced, and corrected fluorescence values were calculated. Standards were used to calculate the accumulated amounts over time and plotted against time. The apparent permeability coefficient (*Papp*) was calculated, according to Equation (1), where *dQ*/*dt* (µg/s) is the dextran/LY flux (liner range) across the barrier, *A* is the insert membrane area (cm^2^), and *C*_0_ is the initial dextran/LY concentration (µg/cm^3^).

(1) $$Papp=\frac{dQ}{dt}\times \frac{1}{A\times C_{0}}$$

### 4.15. Data Analysis

The data were analyzed with GraphPad Prism (version 10.0.3). When comparing healthy vs. PD LRRK2, unpaired *t*-test was used. When comparing exposures, a two-way ANOVA with Tukey’s multiple comparison test was used. Levels of significance * *p* < 0.05; ** *p* < 0.01; *** *p* < 0.001 were used. Outliers were tested with the GraphPad Grubbs’ test.

The visualization of RNA transcriptomic data was carried out by using SRplot, a free online platform for data analysis and visualization [71]. Images were modified with Inkscape 1.2.

## 5. Conclusions

Overall, LRRK2 G2019S causes alterations in hiPSC-derived ECs. These include increased α-synuclein expression, decreased mitochondrial respiration, and altered transcriptome profile, especially the upregulation of fatty-acid-related pathways and altered responses to TNFα.

## Data Availability

The datasets generated during the current study are available from the corresponding author upon reasonable request through Zenodo (doi: 10.5281/zenodo.10809110 (H1), 10.5281/zenodo.10817195 (H2), 10.5281/zenodo.10821851 (H3), 10.5281/zenodo.10829887 (PD1), 10.5281/zenodo.10837057 (PD2), 10.5281/zenodo.10837072 (PD3).

## Supplementary Materials

The following supporting information can be downloaded at: https://www.mdpi.com/article/10.3390/ijms252312874/s1.
