# Supplementary material for: LRRK2 G2019S Mutated iPSC-Derived Endothelial Cells Exhibit Increased α-Synuclein, Mitochondrial Impairment, and Altered Inflammatory Responses
Source: Int J Mol Sci. 2024 Nov 29;25(23):12874. doi: 10.3390/ijms252312874 (PMC11641647; doi:10.3390/ijms252312874)
Supplement: Supplementary file 1 [file ijms-25-12874-s001.zip › ijms-3269350-supplementary.pdf]

Supplementary information

Table S1. Information of the patient

Cell line	Sex	Sample collection at age (years)	Genotype	Status	Sample type	Reference
H1	F	Adult	Normal	Normal	Skin biopsy	Takara Bio (Y00270)
H2	M	62	Normal	Normal	Skin biopsy	Takara Bio (Y00300)
H3	M		Normal	Normal		Takara Bio (Y00320)
H4	M		Normal	Normal		Holmqvist et al [66]
H5	F		Normal	Normal		Holmqvist et al [66]
PD 1	M	64	LRRK2	Parkinson´s disease	Skin biopsy	Holmqvist et al [66]
PD 2	M	55-59	LRRK2	Parkinson´s disease		EBiSC
PD 3	F	77	LRRK2	Parkinson´s disease		EBiSC
PD 3 iso	F	77	LRRK2 corrected	Normal		EBiSC

Table S2. Information of cell lines used in different assays

Protocol 1

Line	qPCR	WB LRRK2	RNAseq	ELISA	Seahorse	LDH	MTT	WB p53	WB CytC	CBA	ICC	Perm (LY)	Perm (Dex)
H1	x	x	x	x	x	x	x	x	x	x	x	x	x
H2	x	x	x	x	x	x	x			x	x	x	x
H3	x	x	x	x	x	x	x	x	x	x	x	x	x
H4				x	x								x
H5 clone 1													
H5 clone 2		x		x	x	x	x	x	x	x	x	x	x
ISO		x		x	x					x	x	x	x
PD1 clone 1	x	x	x	x	x	x	x	x	x	x	x	x	x
PD1 clone 2				x	x			x	x			x	x
PD2	x	x	x	x	x	x	x	x	x	x	x	x	x
PD3	x	x	x	x	x	x	x			x	x	x	x

Protocol 2

Line	ELISA	Seahorse
H4	x	x
H5 clone 1	x	
H5 clone2	x	x
PD1 clone 2	x	x
PD1 clone 2	x	

Table S3. Information of antibodies used in immunocytochemistry and western blotting

Antibodies used for immunocytochemistry

Type	Antibody	Origin	Vendor	Cat. No.	Dilution	Fixation
Primary	α-synuclein	Mouse	BD Bioscience	610787	1:300	FA/MeOH
	CD31	Mouse	Biolegend	303110	1:50	FA/MeOH
	Claudin5	Mouse	Life Technologies	35-2500	1:100	MeOH
	VE cadherin	Mouse	Santa Cruz	sc-9989	1:100	FA/MeOH
	ZO1	Rabbit	Invitrogen	40-2200	1:200	FA/MeOH
Secondary	Anti-mouse 488	goat	Invitrogen	A11001	1:300	
	Anti-mouse 568	goat	Invitrogen	A11004	1:300	
	Anti-rabbit 488	goat	Invitrogen	A11008	1:300	
	Anti-rabbit 568	goat	Invitrogen	A11011	1:300	

Antibodies used for Western blotting

Type	Antibody	Origin	Vendor	Cat. No.	Dilution	Size
Primary	LRRK2	Rabbit	Abcam	ab133474	1:10,000	270 kda
	p53	Sheep	Enzo	KAP-CC030	1:1000	53 kDa
	β actin	Mouse	Sigma	A5441	1:1000	42 kDa
Secondary	Anti-mouse Cy3	Goat	Invitrogen	PA43009	1:2000	
	Anti-rabbit	Donkey	Invitrogen	A16023	1:2500	
	Anti-sheep	Rabbit	Dako	PO163	1:5000	

Antibodies used for immunocytochemistry

Type	Antibody	Origin	Vendor	Cat. No.	Dilution	Fixation
Primary	α-synuclein	Mouse	BD Bioscience	610787	1:300	FA/MeOH
	CD31	Mouse	Biolegend	303110	1:50	FA/MeOH
	Claudin5	Mouse	Invitrogen	35-2500	1:100	MeOH
	VE cadherin	Mouse	Santa Cruz	sc-9989	1:100	FA/MeOH
	ZO1	Rabbit	Invitrogen	40-2200	1:200	FA/MeOH
Secondary	Anti-mouse 488	goat	Invitrogen	A11001	1:300	
	Anti-mouse 568	goat	Invitrogen	A11004	1:300	
	Anti-rabbit 488	goat	Invitrogen	A11008	1:300	
	Anti-rabbit 568	goat	Invitrogen	A11011	1:300	

Antibodies used for Western blotting

Type	Antibody	Origin	Vendor	Cat. No.	Dilution	Size
Primary	LRRK2	Rabbit	Abcam	ab133474	1:10,000	270 kda
	VE cadherin	Mouse	Santa Cruz	sc-9989	1:500	130 kDa
	ZO1	Rabbit	Invitrogen	40-2200	1:500	225 kDa
	p53	Sheep	Enzo	KAP-CC030	1:1000	53 kDa
	Cytochrome C	Mouse	BD Biosciences	556433	1:1000	15 kDa
	β actin	Mouse	Sigma	A5441	1:1000	42 kDa

Secondary	Anti-mouse Cy3	Goat	Cytiva	PA43009	1:2500
	Anti-mouse	Goat	Sigma	A9044	1:5000
	Anti-rabbit	Donkey	Invitrogen	A16023	1:5000
	Anti-sheep	Rabbit	Dako	PO163	1:5000

Table S4. List of primers used in RT-qPCR

Gene symbol	Gene name	TaqMan® Gene expression assay ID
ACTB	Beta actin	4326315 E
CDH5	Cadherin 5	Hs00174344_m1
CLDN5	Claudin 5	Hs01561351_m1
KLF4	Kruppel-like Factor 4	Hs00358836_m1
MEG3	Meg3	Hs00292028_m1
OCLN	Occludin	Hs00170162_m1
POUF5	Oct-4	Hs00742896_s1
SOX2	Sox-2	Hs04234836_s1
TJP1	Tight junction protein 1	Hs01551861_m1

Table S5. Inflammatory exposures for ECs

Exposure	Duration (hours)	Concentration (ng/ml)	Assays
TNFα	4 h	10	RNA sequencing, CBA, LDH
TNFα+IL-1β	4 h	10+10	RNA sequencing, CBA, LDH
TNFα	12 h	10	Permeability, LDH, CBA, ICC, MTT, WB
TNFα+IL-1β	12 h	10+10	Permeability, LDH, CBA, ICC, MTT
TNFα	24 h	10	Permeability, LDH, CBA, ICC, MTT
TNFα+IL-1β	24 h	10+10	Permeability, LDH, CBA, ICC, MTT

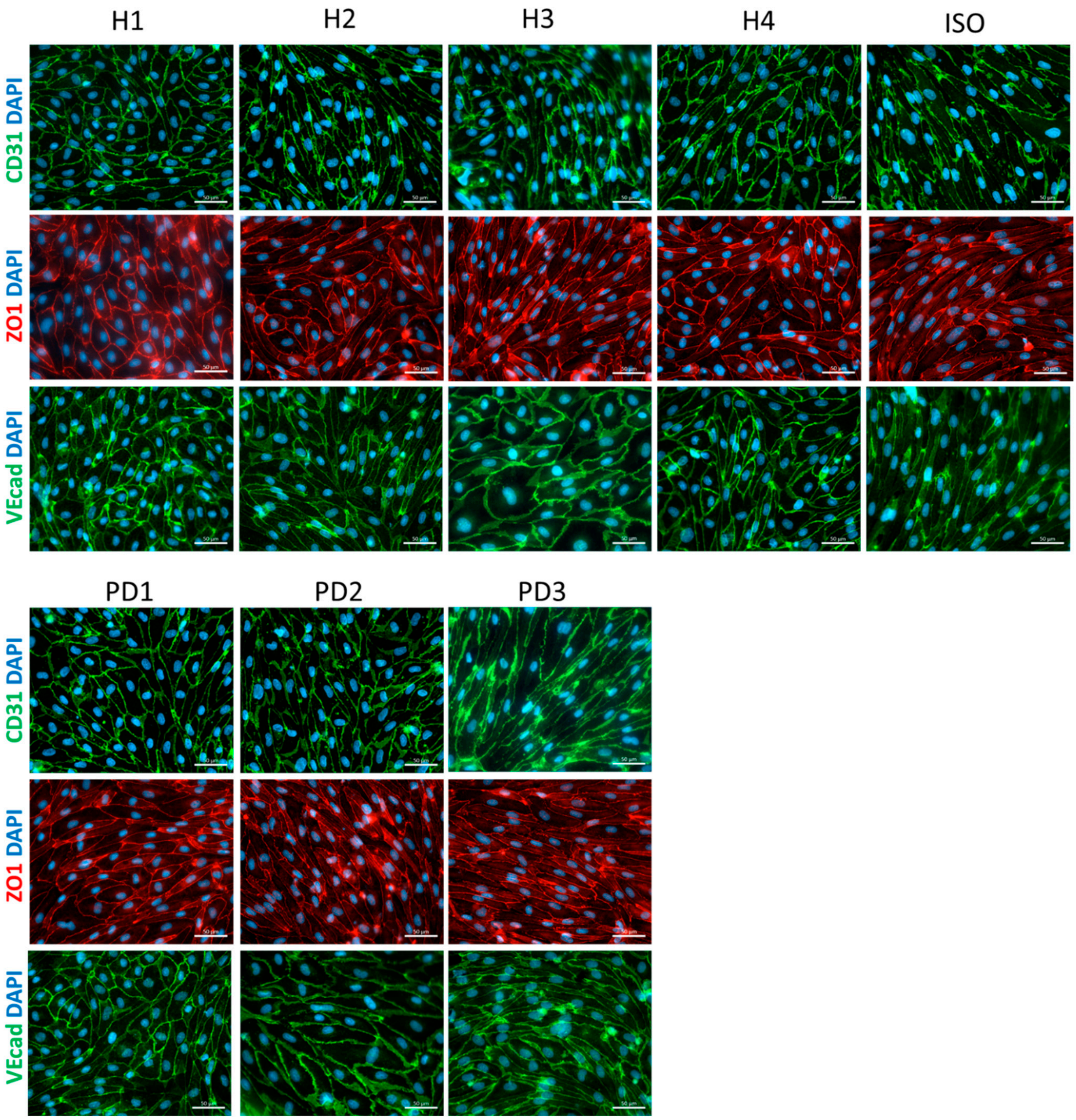

Figure S1. ***Expression of EC markers in hiPSC-derived ECs.*** *Representative immunofluorescence images of healthy (H1-H4, Isogenic) and PD LRRK2 (PD1-3) ECS stained for CD31, ZO1, and VE cadherin. Nuclei stained with DAPI. Scale bar 50 μm.*

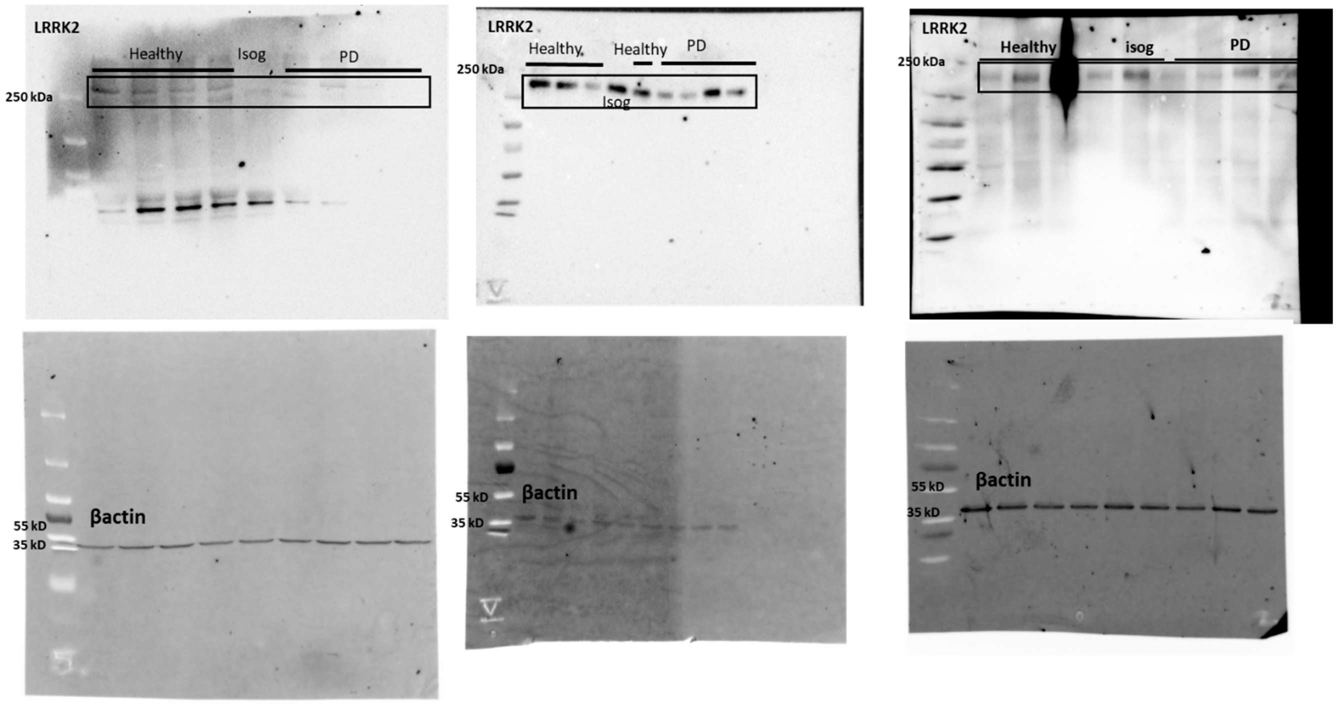

Figure S2. ***Full length Western blot images of LRRK2 and βActin in PD and healthy ECs.*** *n=12 (healthy), 3 (isogenic), 12 (PD LRRK2).*

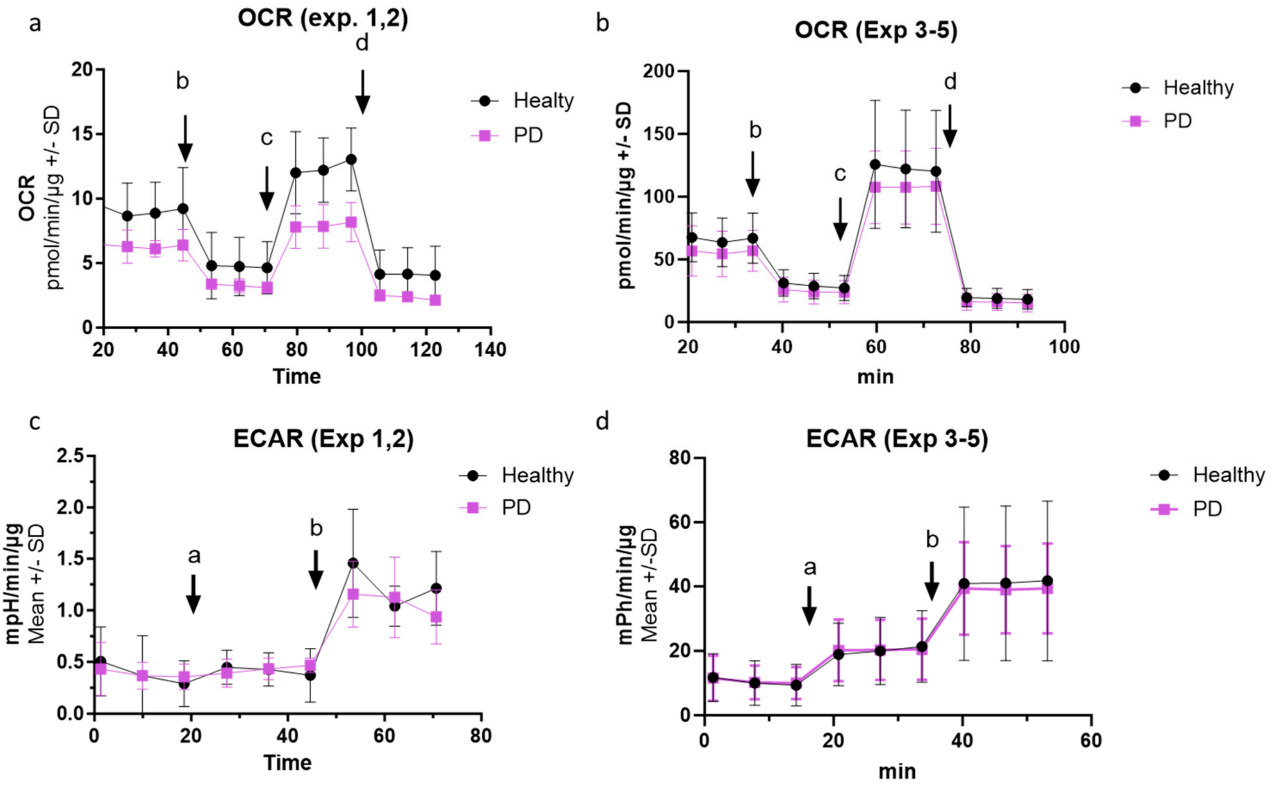

Figure S3. Oxygen consumption rate (OCR) and Extracellular acidification rate (ECAR) traces measured with Seahorse XF24 (a, c) and XF96 (b, d) following additions of 10 µM glucose (A), 1 µM oligomycin (B), 1 µM FCCP (C), and 1 µM antimycin A and rotenone (D). n= 11-12 (healthy), 1 (isogenic), 10-11 (PD LRRK2) from five independent experiments.

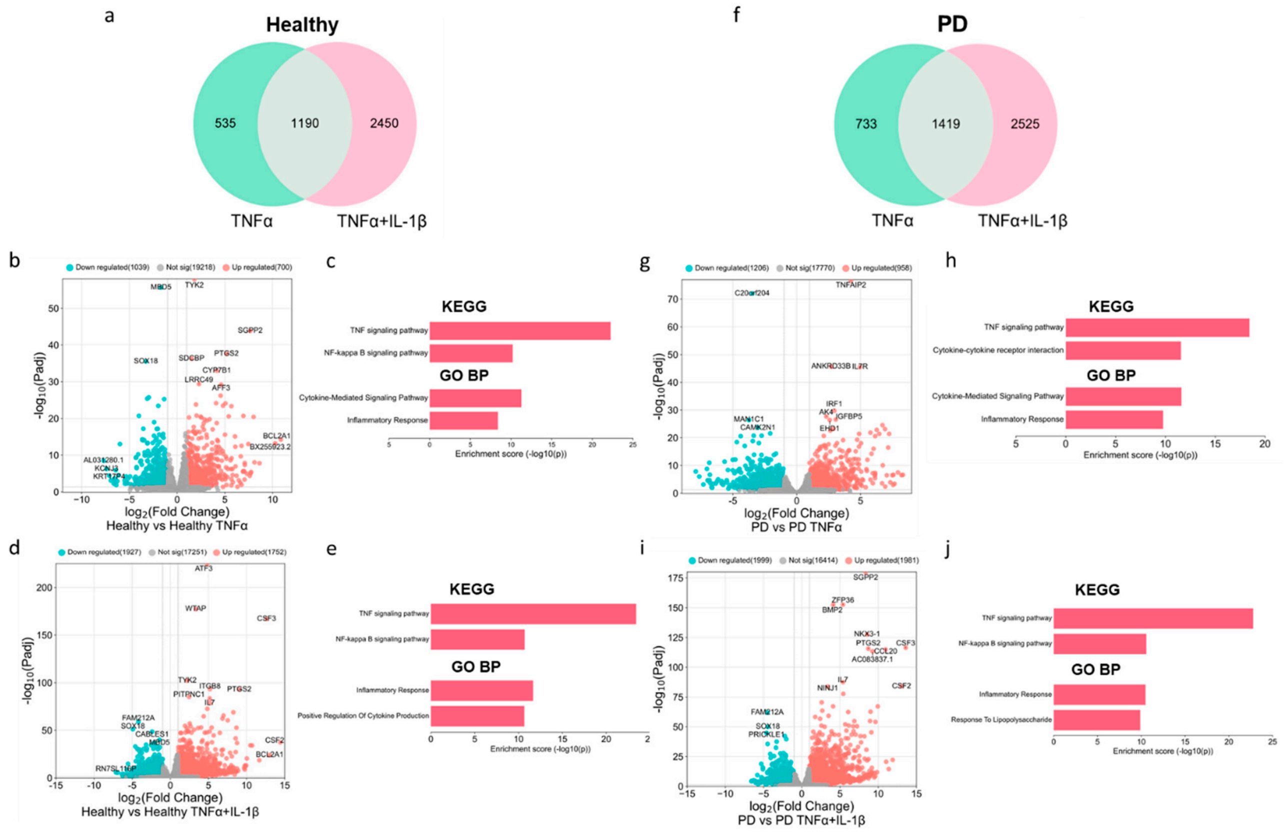

Figure S4. ***Endothelial cells respond to inflammatory stimuli****.* *(A) Venn diagram of DEGs between TNFα and TNF+IL-1β exposed healthy ECs. (B, D) Volcano plot showing up- and down-regulated DEGs in healthy ECs after TNFα (B) or TNFα+IL-1β exposure (D). Adjusted p-value (<0.05) and absolute log2 Fold >1. (C, E) KEGG and GO pathways related to upregulated DEGs in TNFα (C) or TNFα+IL-1β exposed healthy ECs (E). (F) Venn diagram of DEGs between TNFα and TNF+IL-1β exposed PD ECs (G, I) Volcano plot showing up- and downregulated DEGs in PD ECs after TNFα (G) or TNFα+IL-1β exposure (I). Adjusted p-value (<0.05) and absolute log2 Fold >1. (H, J) KEGG and GO pathways related to upregulated DEGs in TNFα or TNFα+IL-1β exposed PD ECs. All exposures lasted for 4 h and concentrations were: 10 ng/ml TNFα or 10 ng/ml TNFα+ IL-1β.*

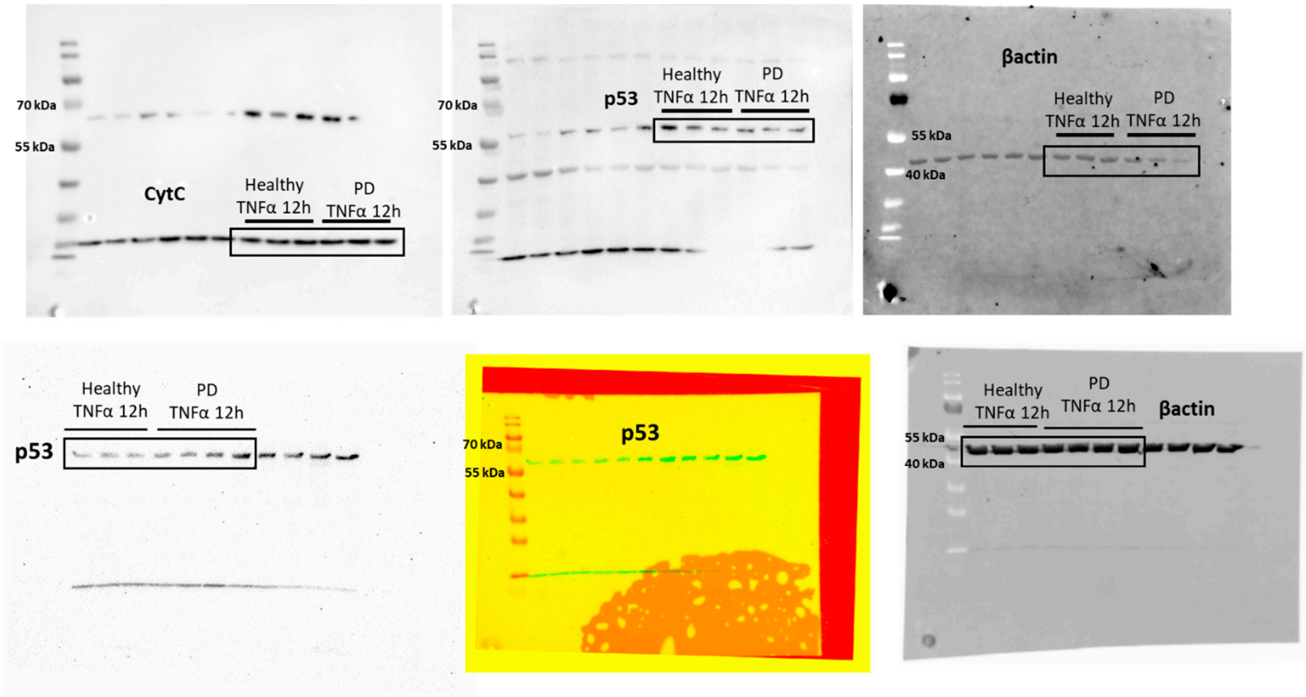

Figure S5. ***Full length Western blot images of Cytochrome C (CytC), p53 and βActin in PD and healthy ECs.*** *n=3 (healthy), 3 (PD LRRK2).*

References

66. Holmqvist, S.; Lehtonen, Š.; Chumarina, M.; Puttonen, K.A.; Azevedo, C.; Lebedeva, O.; Ruponen, M.; Oksanen, M.; Djelloul, M.; Collin, A.; et al. Creation of a Library of Induced Pluripotent Stem Cells from Parkinsonian Patients. *npj Parkinson's Disease* **2016**, *2*, 1–10, doi:10.1038/npjparkd.2016.9.
